# High protein consumption in trained women: bad to the bone?

**DOI:** 10.1186/s12970-018-0210-6

**Published:** 2018-01-31

**Authors:** Jose Antonio, Anya Ellerbroek, Cassandra Evans, Tobin Silver, Corey A. Peacock

**Affiliations:** Department of Health and Human Performance, Nova Southeastern University, 3401 South University Drive, Davie, FL 33328 USA

**Keywords:** DXA, T score, Bone mineral density, Body composition, Protein

## Abstract

**Background:**

It has been posited that the consumption of extra protein (> 0.8 g/kg/d) may be deleterious to bone mineral content. However, there is no direct evidence to show that consuming a high-protein diet results in a demineralization of the skeleton. Thus, the primary endpoint of this randomized controlled trial was to determine if a high-protein diet affected various parameters of whole body and lumbar bone mineral content in exercise-trained women.

**Methods:**

Twenty-four women volunteered for this 6-month investigation (*n* = 12 control, n = 12 high-protein). The control group was instructed to consume their habitual diet; however, the high-protein group was instructed to consume ≥2.2 g of protein per kilogram body weight daily (g/kg/d). Body composition was assessed via dual-energy x-ray absorptiometry (DXA). Subjects were instructed to keep a food diary via the mobile app MyFitnessPal**®**. Exercise or activity level was not controlled. Subjects were asked to maintain their current levels of exercise.

**Results:**

During the 6-month treatment period, there was a significant difference in protein intake between the control and high-protein groups (mean±SD; control: 1.5±0.3, high-protein: 2.8±1.1 g/kg/d); however, there were no differences in the consumption total calories, carbohydrate or fat. Whole body bone mineral density did not change in the control (pre: 1.22±0.08, post: 1.22±0.09 g/cm^2^) or high-protein group (pre: 1.25±0.11, post: 1.24±0.10 g/cm^2^). Similarly, lumbar bone mineral density did not change in the control (pre: 1.08±0.16, post: 1.05±0.13 g/cm^2^) or high-protein group (pre: 1.07±0.11, post: 1.08±0.12 g/cm^2^). In addition, there were no changes in whole body or lumbar T-Scores in either group. Furthermore, there were no changes in fat mass or lean body mass.

**Conclusion:**

Despite an 87% higher protein intake (high-protein versus control), 6 months of a high-protein diet had no effect on whole body bone mineral density, lumbar bone mineral density, T-scores, lean body mass or fat mass.

## Background

It is has been postulated that high protein consumption (i.e., > RDA) poses several health risks. These could include renal dysfunction as well as bone demineralization [1]. However, there is evidence that consuming a high-protein diet for one year has no negative effect on kidney or liver function [2]. Whether it is also true that bone health is not compromised by a high-protein diet is not entirely certain according to health care professionals. Much of the purported harm caused by dietary protein with regards to bone is often explained via the acid-ash hypothesis. The acid-ash diet hypothesis suggests that acid from a typical Western diet may cause bone demineralization with the consequent excretion of calcium [3]. This in turn may lead to osteoporosis. Thus, protein-containing foods (e.g., meat) provide acid precursors, whereas vegetables (protein) provide base precursors not found in animal foods. However, in one investigation, bone mineral density was not associated with the ratio of animal to vegetable protein intake [4]. Interestingly, 12 weeks of resistance training did not enhance bone formation or inhibit bone resorption in young adult women; subsequently, maintenance of a high protein intake for 10 days in these women also demonstrated no effects on bone metabolism [5]. Observational data suggests that a higher protein intake does not adversely affect bone health in premenopausal women; however, low vegetable protein intake is associated with lower BMD [6]. There are no investigations in exercise-trained women that have examined the effects of a chronic high-protein diet. Thus, the primary purpose of this investigation was to determine if consuming extra protein (≥ 2.2 g/kg/d) affected bone mineral content or density in exercise-trained women.

## Methods

### Participants

Twenty-four exercise-trained female subjects volunteered for this investigation (i.e., had to be performing a minimum of resistance- and/or aerobic-training sessions three times per week for at least the last year). Subjects came to the laboratory on two occasions for testing (baseline and 6 months post). In accordance with the Helsinki Declaration, the university’s Institutional Review Board approved all human subjects procedures. Written informed consent was obtained prior to participation. All testing took place between 1130 and 1400 h.

### Body composition

Subjects had their height and weight determined using a calibrated scale. Body composition (i.e., was assessed with a dual-energy X-ray absorptiometry machine (DXA) (Model: Hologic Horizon W; Hologic Inc., Danbury CT USA). Quality control calibration procedures were performed on a spine phantom. Subjects wore typical athletic clothing and removed all metal jewelry. They were positioned supine on the DXA within the borders delineated by the scanning table. Each whole body scan took approximately seven minutes. In addition, a lumbar spine scan was performed. Subjects were positioned supine on the scanning table with their legs resting on a pad that allowed them to have their knee and hip joint at a 90-degree angle. The lumbar scan took approximately 15 s.

### Diet and exercise

Subjects were randomly assigned to a control or high-protein group. The control group was instructed to maintain their habitual diet. The high-protein group was instructed to consume ≥ 2.2 g/kg/d of protein. Subjects had the choice of consuming more protein via their typical diet (i.e., eat more protein-containing foods) or the consumption of protein powder. Dymatize**®** provided whey and casein protein powder whereas vegan protein powder was provided by MusclePharm**®**. Subjects were also instructed to keep a food log (three times per week) on the MyFitnessPal mobile app. All subjects had prior experience using the mobile app and as such were quite skilled at logging their food intake. The subjects’ training regimen was not altered in any way; thus, each subject self-selected their exercise regimen.

### Statistical analysis

An analysis of variance was used to assess differences between the control and treatment group for measures of bone health and body composition. A paired and independent t-test were utilized to assess any main effects of condition, groups or significant interactions. All data is presented as the mean±SD. Prism 6 software was used for statistical analyses.

## Results

Twenty-four exercise-trained women completed this investigation (mean±SD. Age years: control 36.0±9.5, high-protein 37.3±8.9; Height cm: control 167.5±10.1, high-protein 167.5±5.1). Each subject in the high-protein diet group increased their protein intake primarily via the protein powder that was provided. The majority of our subjects performed both aerobic and resistance exercises 1–3 h per week (i.e., 44–48% of the subjects) (Table 1); however, there was a subset of subjects (36%) that performed a high volume of aerobic training (> 7 h per week). All subjects performed aerobic exercise regularly; however, 12% did not do any resistance training. Moreover, the types of athletes in the study were as follows. The control group was comprised of: 1 world-class distance runner, 4 competitive (state-level) stand-up paddlers, and 7 resistance-trained women. The high-protein group was comprised of: 1 national class distance runner, 1 world-class stand-up paddler, 4 competitive (state-level) stand-up paddlers, 1 competitive cyclist (state-level), and 5 resistance-trained women.

**Table 1:** Hours of Training Per Week

	0 h/wk	1–3 h/wk	4–6 h/wk	> 7 h/wk
Aerobic Training	0%	44%	20%	36%
Resistance Training	12%	48%	32%	8%

For example: 44% of our research subjects performed 1–3 h of aerobic training weekly whereas 48% performed resistance training weekly

There was a significant difference between the high-protein and control group for daily protein intake (Table 2). Even though the total daily caloric intake of the high-protein group exceeded the control by ~ 226 kcals, it was not significantly different (*p* = 0.1710); furthermore, no other differences existed regarding their dietary intake. Moreover, there were no within- or between-group differences in any measure of bone health or body composition (Tables 3 and 4).

**Table 2:** Diet

	Control	High-Protein
Energy (kcal)	1580±277	1877±441
Protein (g)	91±17	169±55*
Carbohydrate (g)	169±53	157±50
Fat (g)	60±8	64±17
Cholesterol (mg)	278±67	547±624
Sodium (mg)	1838±537	2576±1085
Sugar (g)	58±22	52±29
Fiber (g)	20±7	28±16
Energy (kcal/kg/d)	26.1±4.2	30.7±8.1
Protein (g/kg/d)	1.5±0.2	2.8±1.1^#^
Carbohydrate (g/kg/d)	2.8±0.9	2.6±0.8
Fat (g/kg/d)	1.0±0.2	1.0±0.3

Data are expressed as the mean±SD. *n* = 12 for both groups. *p = 0.0001, ^#^*p* = 0.0004 - Denotes statistically significant differences between the control and high-protein group. Legend: *d* day, *g* gram, *kg* kilogram

**Table 3:** Bone

	Control Pre	Control Post	High-Protein Pre	High-Protein Post
Bone Mineral Content (kg)	2.47±0.35	2.47±0.34	2.55±0.38	2.53±0.40
Bone Mineral Density (g/cm^2^)	1.22±0.08	1.22±0.09	1.25±0.11	1.24±0.10
Total Body T-Score	1.4±1.0	1.3±1.1	1.7±1.3	1.7±1.3
Lumbar Bone Mineral Content (grams)	65.8±16.1	64.5±10.3	69.6±8.9	71.6±9.8
Lumbar Bone Mineral Density (g/cm^2^)	1.08±0.16	1.05±0.13	1.07±0.11	1.08±0.12
Lumbar T-Score	0.3±1.5	0.0±1.1	0.2±1.0	0.3±1.1

Data are expressed as the mean±SD. n = 12 for both control and high-protein groups. There were no significant differences within or between groups. Legend: *cm* centimeter, *g* grams, *kg* kilograms. The T-score is the number of standard deviations that one’s bone mineral density is above or below the average. Scale for T-score: −1 and above is normal. Between −1 and −2.5 is osteopenia. -2.5 or below is osteoporosis

**Table 4:** Lean Body Mass and Fat Mass

	Control Pre	Control Post	High-Protein Pre	High-Protein Post
Body Weight (kg)	61.9±10.7	62.5±10.2	61.5±5.8	61.8±6.5
Lean Body Mass (kg)	42.2±6.9	42.9±6.3	44.3±5.1	44.5±5.3
Fat Mass (kg)	17.2±5.2	17.1±6.0	14.7±1.8	14.7±2.2
Body Fat Percentage (%)	27.6±5.6	27.0±6.8	24.0±3.3	23.9±3.4

Data are expressed as the mean±SD. n = 12 for both groups. There were no significant differences within or between groups. Legend: *kg* kilograms

## Discussion

This is the first investigation to examine the effects of a chronic high-protein diet (2.8 g/kg/d) in exercise-trained women. In the current study, the high-protein group consumed 87% more protein than the control. The ~ 226 kcal difference in energy intake (high-protein > control) is largely due to the increase in protein consumption. It should be noted that the high-protein group exhibited a wide variation in intake (mean±SD 169±55 g/daily). This was largely due to one subject that consumed in excess of 4 g/kg/d of protein. Nevertheless, in spite of a significantly higher protein intake, there were no changes in bone mineral density, bone mineral content (i.e., whole body, lumbar spine) or T-score. There have been other investigations that have made direct measures of bone health after the consumption of a higher protein diet. Kenny et al. evaluated the effect of a 1-year treatment that consisted of dietary soy protein and/or soy isoflavones on bone health in late postmenopausal women [7]. They found that neither soy protein nor isoflavones (in combination or alone) had any effect on BMD. It should be noted that the protein intake from that study was actually quite low (~ 0.9 g/kg/d) [7]. Ballard et al. conducted a 6-month investigation on protein supplementation and bone health [8]. They discovered that additional protein had no effect on BMD or bone size in young adults (18–25 years). It should be noted that the protein intake of the subjects in the Ballard et al. investigation was quite low as well (1.0–1.2 g/kg/d). Moreover, in older women and men (> 60 years), whey protein supplementation (total daily protein intake of 1.0–1.1 g/kg/d) had no effect on bone mass; however, it did increase truncal lean mass after an 18-month treatment period [9].

It must be acknowledged that the protein intake of the aforementioned studies was quite low. Athletes or individuals that exercise regularly are often advised to consume at least twice the recommended daily allowance (RDA) of protein [10–14]. The protein intake in our investigation was 1.5 and 2.8 g/kg/d for the control and high-protein groups. It should be emphasized that the protein intake from our control group exceeded those of other studies [7–9]. So perhaps in order to observe a change in bone parameters, a much higher dose is needed. Our high-protein group consumed protein at a dose 2.5 times greater than the RDA. Thus, if there were a deleterious effect of protein consumption, one would reasonably expect to see this at such a high dose. On the contrary, our investigation found no effect on bone mineral content or density.

This is in agreement with other studies that have utilized moderate to high protein intakes. Cao et al. provided post-menopausal women with a diet of 1.7 g/kg/d of protein for 7 weeks and found no adverse effects on bone health [15]. Ballard had young (18–25 years) subjects consume 2.2 g/kg/d of protein over a 6-month treatment period [8]. Although they did not measure bone mass directly, they discovered that biomarkers for bone formation were elevated (e.g., IGF-1). Moreover, in the presence of high calcium intake, consuming a high-protein diet (2.5 g/kg/d) for one month in hyperlipidemic men and women (56 years) did not have a negative effect on calcium balance [16]. However, the treatment period was rather short and they did not make any direct measures of bone mass.

Our investigation is the first to use exercise-trained women. This is significant in that it is exercise-trained individuals that purposefully consume a higher protein diet [2, 10, 11, 13, 17–23]. Sedentary individuals do not typically consume a high-protein diet. And it is clear that a very high intake of protein (2.5 x greater than the RDA) does not have a negative effect on bone mineral content or density.

Another interesting find in our investigation was that despite consuming more calories per day, the high-protein group did not experience a change in fat mass. This supports work from other investigations [10, 24–26]. In fact, when combined with a change in training, a higher protein diet can promote a loss of fat mass [10]. It is unclear why fat mass might decrease in response to protein overfeeding combined with a change in one’s training program. Perhaps it is non-exercise activity thermogenesis or diet-induced thermogenesis with increased protein consumption [27, 28]. Also, animal data suggests that a high-protein diet might reduce fat mass by inhibiting lipogenesis in the liver [29]. Nevertheless, the current investigation demonstrated that body composition as well as bone mass does not change unless in the absence of alterations in the exercise stimulus.

It should be noted that the total energy intake of our subjects seemed rather low for active individuals. For instance, the control and high-protein groups consumed on average 1580 and 1877 cal per day. A simple estimation of basal metabolic rate using the Harris Benedict equation would suggest that at minimum these individuals should be consuming greater than 2000 cal per day [30]. Thus, it is likely that our subjects were underreporting total energy intake, particularly from carbohydrate and fat. It is however plausible that protein intake was more accurately estimated inasmuch as subjects derived the added protein from protein powder sources. Nonetheless, despite the drawbacks of using dietary records, it is evident that extra protein supplementation has no harmful effect on bone mineral content or density.

## Conclusion

In conclusion, exercise-trained female subjects that consume a diet that is 2.5 times greater than the RDA for protein experience no harmful effects on bone mineral density or content. Thus, there is no evidence that a high protein intake causes harm to bone health.
